# Supplementary material for: Endogenous and Exogenous Modulation of Nrf2 Mediated Oxidative Stress Response in Bovine Granulosa Cells: Potential Implication for Ovarian Function
Source: Int J Mol Sci. 2019 Apr 2;20(7):1635. doi: 10.3390/ijms20071635 (PMC6480527; doi:10.3390/ijms20071635)
Supplement: Supplementary file 1 [file ijms-20-01635-s001.pdf]

Supplementary Materials:

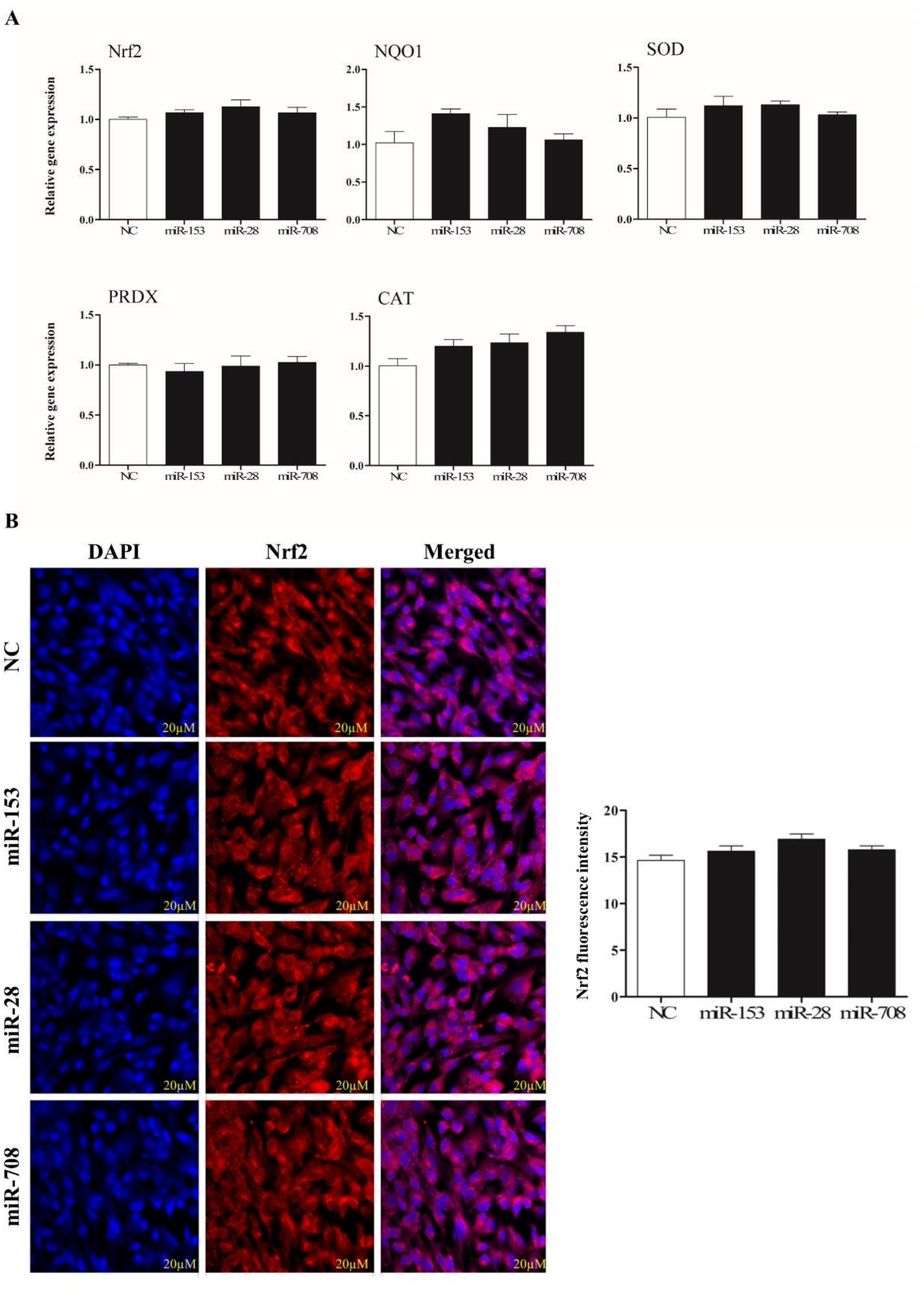

Figure S1. Quantitative RT-PCR of Nrf2 and its downstream antioxidant targets in bovine granulosa cells transfected with miR-153, miR-28 and miR-708 inhibitors (**A**). Immunocytochemistry of Nrf2 in bovine granulosa cells transfected with candidate miRNAs inhibitors (**B**). White bars represent granulosa cells transfected with inhibitor negative control (NC) and dark bars represent cells transfected with miRNA inhibitors. Data are presented as mean ± SEM of three independent biological replicates. Bars with different letters (a,b) showed statistically significant differences ($p<0.05$).

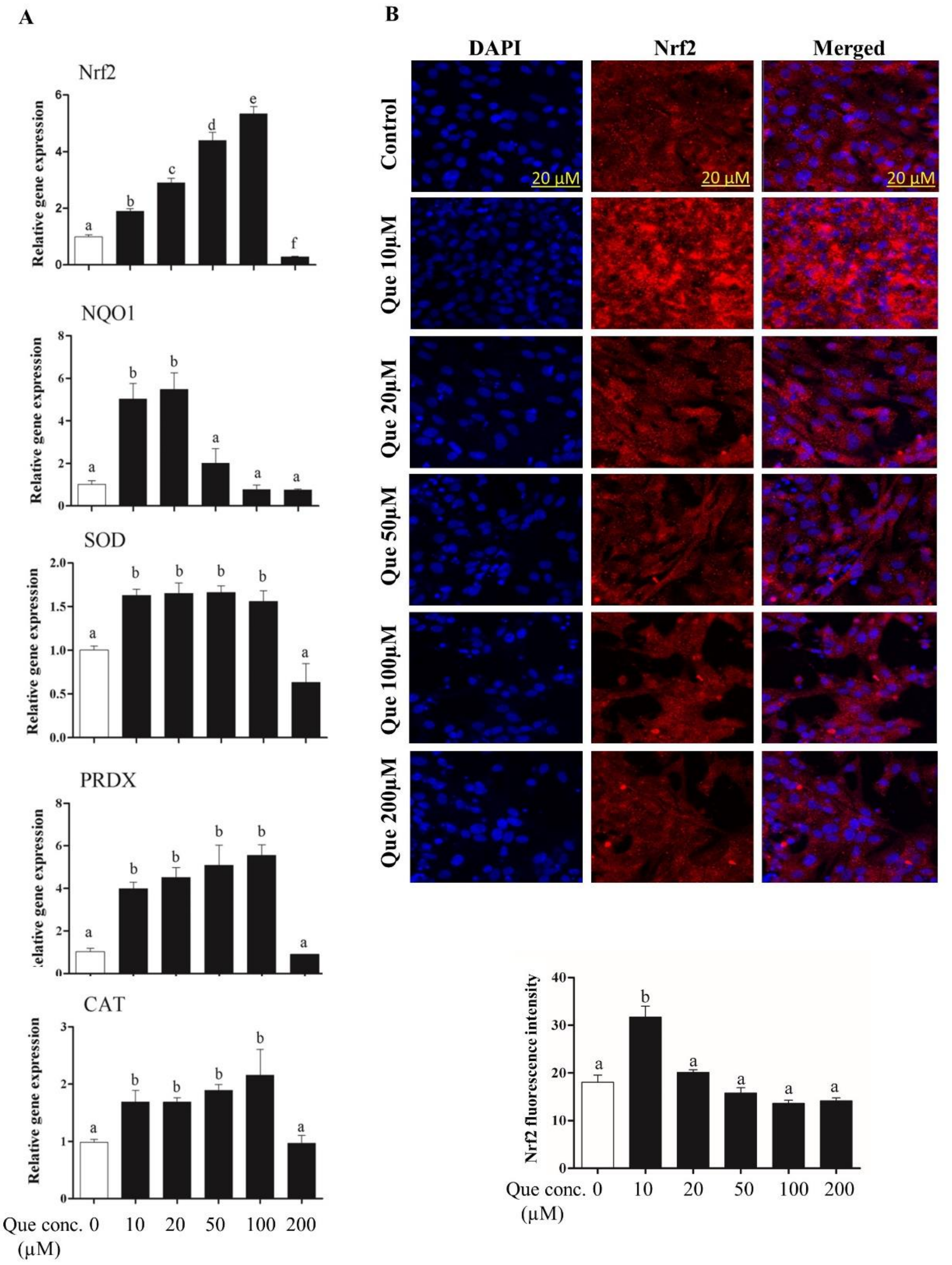

Figure S2. Quercetin modulates the mRNA expression level of Nrf2 and its downstream antioxidants. Quantitative RT-PCR of Nrf2 and its down stream antioxidants in bovine granulosa cells control group (white bar) or different Quercetin concentrations (black bar) (**A**). Immunocytochemistry of Nrf2 in bovine granulosa cells co-incubated with different Que concentration (0 – 200 µM) (**B**). Data are presented as mean ± SEM of three independent biological replicates. Bars with different letters (a,b,c,d,e,f) showed statistically significant differences ($p<0.05$).

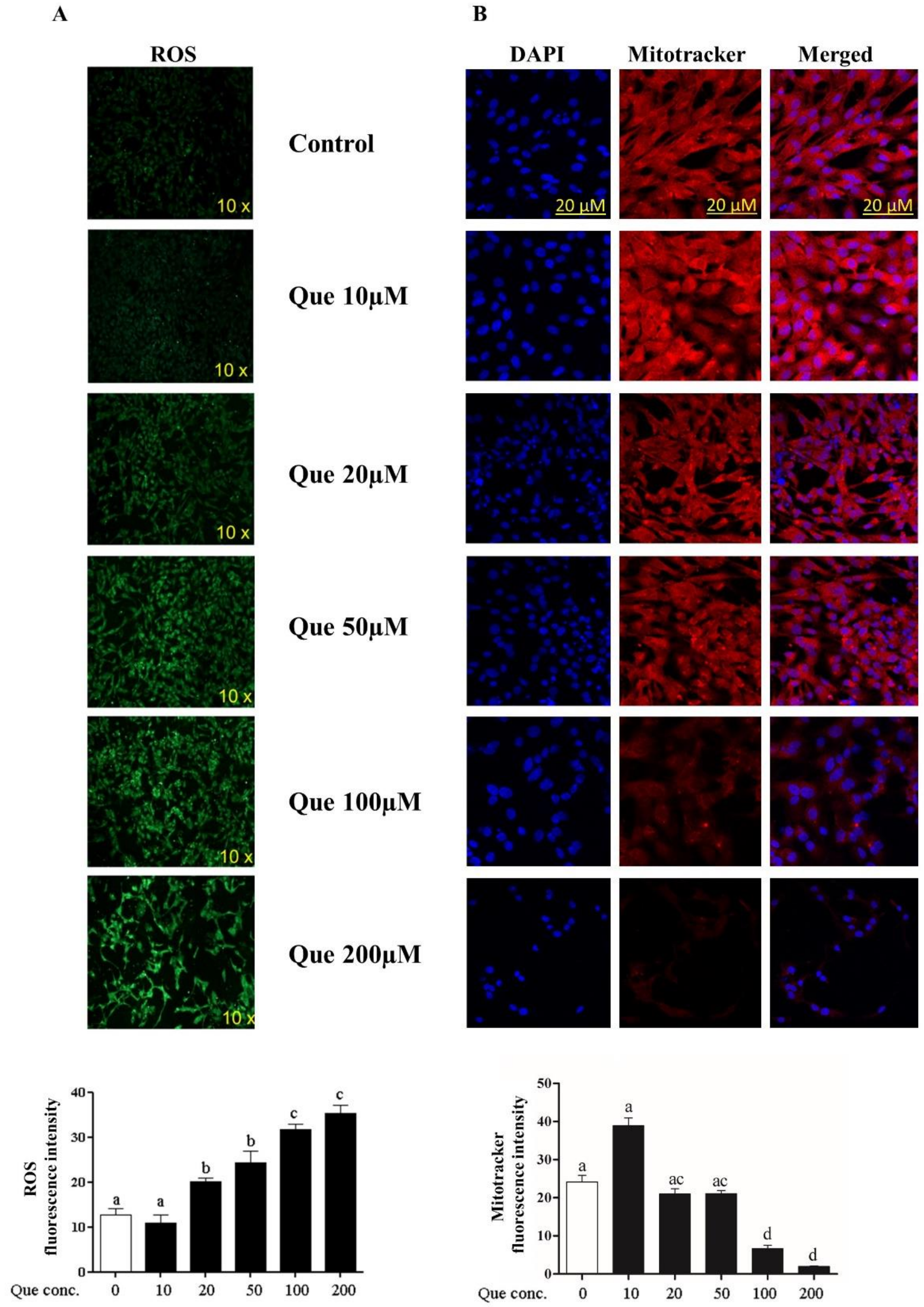

Figure S3. Quercetin increases intracellular ROS accumulation levels in dose dependent manner (**A**). Supplementation of quercetin at 10μM increased mitochondrial activity. However, higher concentration of quercetin reduced the mitochondrial activity (**B**). Data are presented as mean ± SEM of three independent biological replicates. Bars with different letters (a,b,c,d) showed statistically significant differences ($p<0.05$)

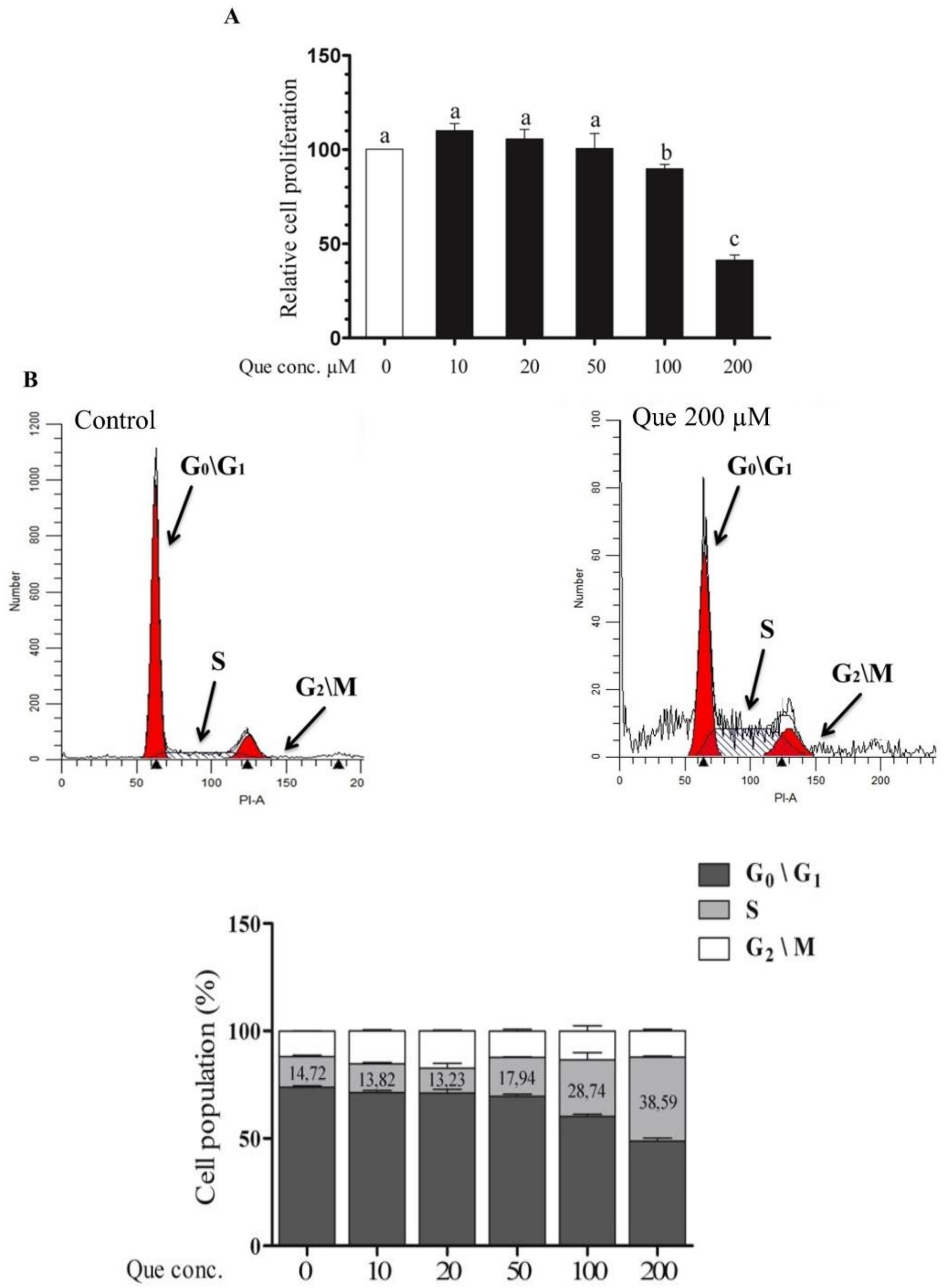

Figure S4. Supplementation of higher concentrations of quercetin resulted in reduced bovine proliferation of granulosa cells (**A**). Flow cytometry analysis showed a shift in cell cycle transition towards the S-phase (**B**). Data are presented as mean ± SEM of three independent biological replicates. Bars with different letters (a,b) showed statistically significant differences ($p<0.05$)

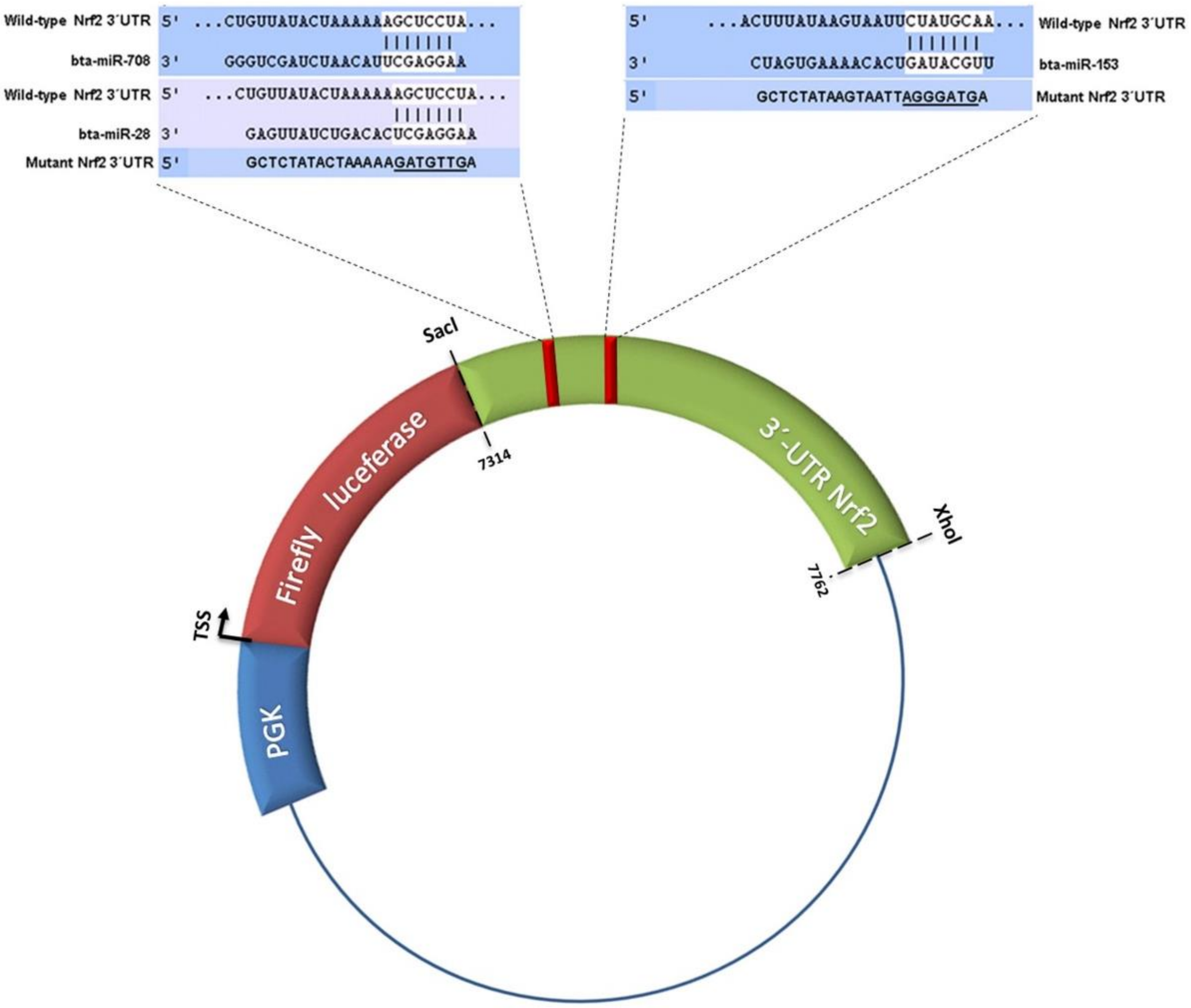

Figure S5. MiRNAs coordinately target Nrf2 mRNA in bovine granulosa cells. Putative binding sites of miR-153, miR-28 and miR-708 and their genomic coordinates in the 3´-UTR of bovine Nrf2 mRNA are indicated . Plasmid with wild-type and mutant sequences (underlined) for the miRNA binding were fused into the downstream multiple cloning sites of the firefly luciferase gene between the SacI and XhoI restriction sites. The PGK upstream promoter and transcription start site (TSS) are indicated in blue and arrow, respectively.

Table S1: The list of primers and their sequences of selected candidate genes used for qRT-PCR analysis

Gene	Primer sequence	Size (bp)	Accession number
**GAPDH**	F: 5′-ACCCAGAAGACTGTGGATGG-3′<br>R: 5′-ACGCCTGCTTCACCACCTT-3′	247	NM_001034034
**Β-ACTIN**	F: 5′-GGCATTCACGAAACTACCTT-3′<br>R: 5′-CAATCCACACGGAGTACTTG-3′	208	NM_173979
**Nrf2**	F: 5′-CCCAGTCTTCACTGCTCCTC-3′<br>R: 5′-TCAGCCAGCTTGTCATTTTG-3′	165	NM_001011678
**NQO1**	F: 5′-AACCAACAGACCAGCCAATC-3′<br>R: 5′-CACAGTGACCTCCCATCCTT-3′	154	NM_001034535.1
**SOD1**	F: 5′-TGCCATCGTGGATATTGTAG-3′<br>R: 5′-GCAATTCCAATTACACCACA-3′	174	NM_174615
**PRDX1**	F: 5′-TGGATCAACACACCCAAGAA-3′<br>R: 5′-GTCTCAGCGTCTCATCCACA-3′	217	NM_174431.1
**CAT**	F: 5′-TGGGACCCAACTATCTCCAG-3′<br>R: 5′-AAGTGGGTCCTGTGTTCCAG-3′	178	NM_001035386.1
